# Bis(nitrato-κ*O*)(1,4,8,11-tetra­aza­cyclo­tetra­decane-κ^4^ *N*)zinc(II) methanol monosolvate

**DOI:** 10.1107/S2414314622008549

**Published:** 2022-08-31

**Authors:** Yoshimi Ichimaru, Koichi Kato, Masaaki Kurihara, Wanchun Jin, Tohru Koike, Hiromasa Kurosaki

**Affiliations:** a Faculty of Pharmaceutical Sciences, Shonan University of Medical Sciences, 16-48 Kamishinano, Totsuka-ku, Yokohama, Kanagawa 244-0806, Japan; b College of Pharmacy, Kinjo Gakuin University, 2-1723 Omori, Nagoya 463-8521, Japan; c Department of Functional Molecular Science, Institute of Biochemical & Health Sciences, Hiroshima University, 1-2-3 Kasumi, Minami-ku, Hiroshima 734-8553, Japan; Vienna University of Technology, Austria

**Keywords:** crystal structure, zinc(II) complex, cyclam

## Abstract

The two Zn^II^ atoms in the crystal structure of the title complex, [Zn(NO_3_)_2_(C_10_H_24_N_4_)]·CH_3_OH, have a distorted octa­hedral coordination sphere, defined by 1,4,8,11-tetra­aza­cyclo­tetra­decane (cyclam) N atoms in the equatorial plane and nitrate O atoms in the axial sites. The conformation of the cyclam is *trans*-III (*R*, *R*, *S*, *S*), which is typical for metal–cyclam complexes. Nitrate anions are involved in intra- and inter­molecular hydrogen bonding with the N–H groups of the Zn^II^–cyclam unit. Together with the methanol solvent mol­ecule, the hydrogen-bonding network connects the Zn^II^–cyclam units into ribbons running parallel to the *a* axis.

## Abstract 2

The coordination of the central Zn^II^ atoms in the two different Zn-cyclam units is distorted octa­hedral.

## Graphical abstract

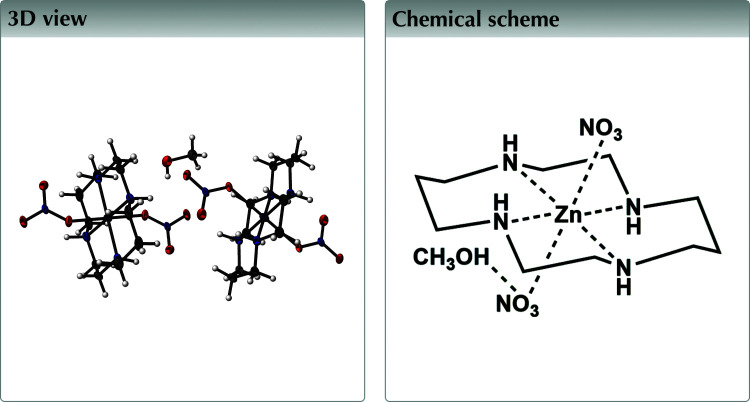

## Structure description

Cyclam is a well-known macrocyclic polyamine and water-soluble ligand that can strongly chelate transition-metal cations. As a result, various cyclam derivatives and metal complexes have been synthesized, and their crystal structures have been described. The crystal structure of the title zinc nitrate complex, on the other hand, is the first reported in this context. We anti­cipate that, in future, this structural property can be used in the development of new functional materials.

**Figure 1:**
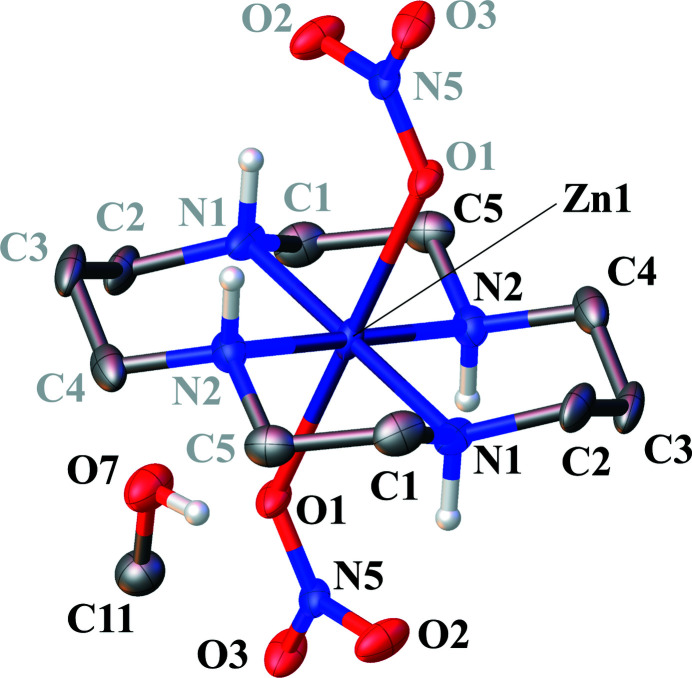
The Zn^1I^–cyclam complex involving Zn1 and the methanol solvate mol­ecule. Displacement ellipsoids are drawn at the 50% probability level;. C-bound H atoms were omitted for clarity. Gray atom labels represent atoms generated by symmetry expansion (symmetry operation: 2 − *x*, 1 − *y*, 1 − *z*).

**Figure 2:**
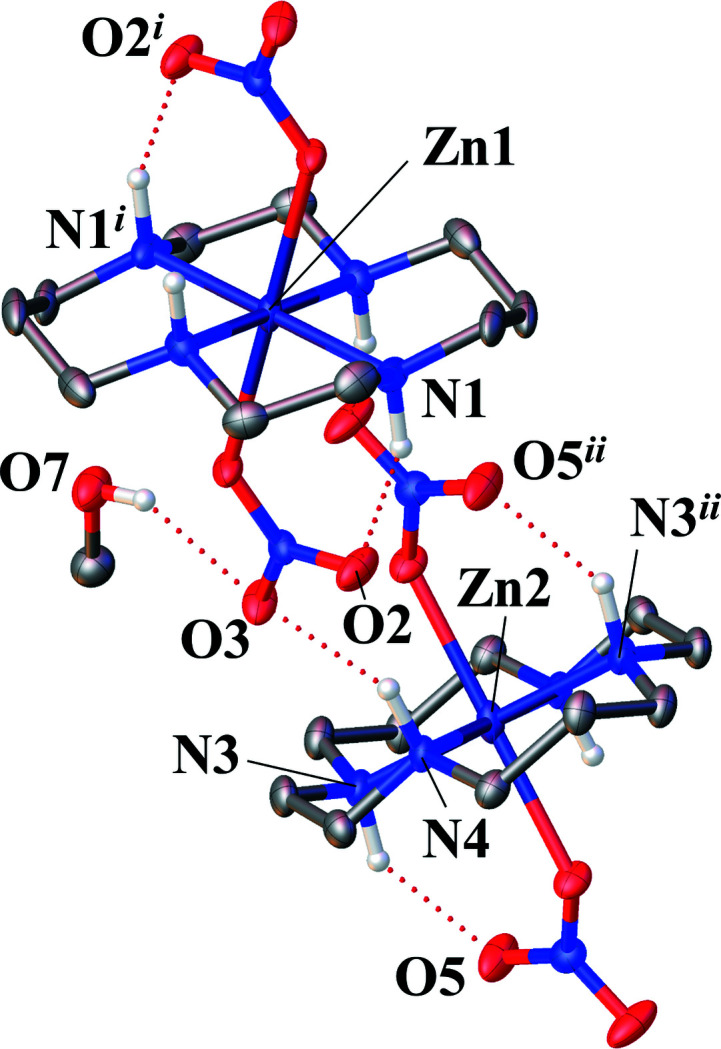
The hydrogen-bonding network between Zn^II^–cyclam complexes with displacement ellipsoids drawn at the 50% probability level. C-bound H atoms were omitted for clarity. Hydrogen-bonding inter­actions are shown as dotted lines. [Symmetry codes: (i) 2 − *x*, 1 − *y*, 1 − *z*; (ii) 1 − *x*, 1 − *y*, 1 − *z*].

The asymmetric unit of the title complex, [Zn^II^(C_10_H_24_N_4_ = cyclam)](NO_3_)_2_·CH_3_OH, comprises two half-Zn^II^–cyclam complexes that are centered on Zn1 and Zn2, as well as two nitrate anions that coordinate to each Zn^II^ atom, and a methanol solvent mol­ecule. The two half-Zn^II^–cyclam complexes are completed by inversion symmetry. Each Zn^II^ atom is coordinated in a planar fashion by the four N atoms of the cyclam ligand. N1, N2, N1^i^, and N2^i^ [symmetry code: (i) 2 − *x*, 1 − *y*, 1 − *z*] define the cyclam plane around Zn1, and nitrate atoms O1 and O1^i^ coordinate at the axial positions of the resulting distorted octa­hedron (Fig. 1 ▸). For Zn2, the equatorial plane is defined by N3, N4, N3^ii^, and N4^ii^ [symmetry code: (ii) 1 − *x*, 1 − *y*, 1 − *z*], and the axially bound O atoms by O4 and O4^ii^ (Fig. 2 ▸). The coordination environments of the two central Zn^II^ atoms are similar to that of Co(cyclam)Cl_2_ (Oba & Mochida, 2015 ▸). The conformation of the cyclam structure is *trans*-III (*R*, *R*, *S*, *S*) type, which is the most energetically favorable conformation (Bosnich *et al.*, 1965 ▸). The conformation is generally consistent with previous reports for metal–cyclam complexes such as Cu^II^ (Emsley *et al.*, 1990 ▸), Ni^II^ (Prasad *et al.*, 1987 ▸), and Pd^II^ (Hunter *et al.*, 2004 ▸). The Zn1—O1 and Zn2—O4 bond lengths are 2.3045 (18) and 2.3233 (19) Å, respectively, which is longer than in the Zn^II^–nitrate ion (*ca* 2.0 Å; Ichimaru *et al.*, 2021 ▸; Kinoshita-Kikuta *et al.*, 2021 ▸), owing to the hydrogen-bonding network detailed below. The N1—Zn1—O1 and N2—Zn1—O1 bond angles are 92.98 (8)° and 89.14 (9)°, and N3—Zn2—O4 and N4—Zn2—O4 are 91.98 (8) and 87.95 (9)°. These angles imply that both Zn^II^ atoms are on the centroid of the plane created by the four cyclam N atoms. However, the two cyclam rings chelating Zn1 and Zn2 have different asymmetric structures: N1—H1 and N2—H2 have *syn*-configurations, while N3—H3 and N4—H4 have *anti*-configurations.

**Figure 3:**
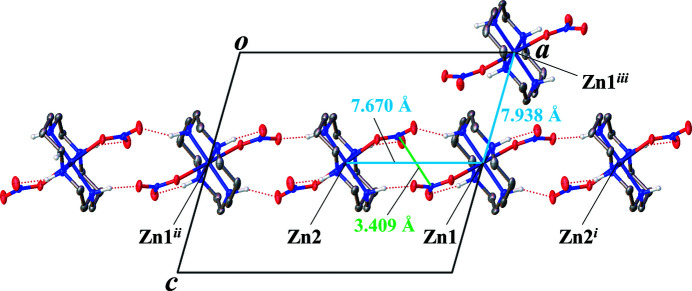
Packing view down the *b* axis of the title complex with displacement ellipsoids drawn at the 50% probability level. Solvent mol­ecules and C-bound H atoms were omitted for clarity. Hydrogen-bonding inter­actions are shown as dotted lines. [Symmetry codes: (i) 2 − *x*, 1 − *y*, 1v*z*; (ii) 1 − *x*, 1 − *y*, 1 − *z*; (iii) *x*, 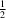 − *y*, − 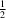 + *z*].

In addition to the methanol solvate mol­ecule, two nitrate anions are involved in the formation of an inter- and intra­molecular hydrogen-bonding network. The nitrate anion coordinating to Zn1 forms an intra­molecular hydrogen bond (O2⋯H1—N1) and an inter­molecular hydrogen-bond (O3⋯H4—N4) (Fig. 2 ▸). N2—H2 and N3—H3 create hydrogen bonds with the other nitrate ion. As a result, the hydrogen-bond network includes all N-bound H atoms. Table 1 ▸ summarizes numerical data of the hydrogen bonding. In the crystal packing, the different moieties form ribbons parallel to the *a* axis through the hydrogen-bonding network (Fig. 3 ▸). The distances between Zn atoms parallel to the *a* axis, for example, Zn1⋯Zn2, are 7.6706 (3) Å (Fig. 3 ▸). The distances between Zn atoms in neighboring ribbons, for example, Zn1⋯Zn1^iii^ [symmetry code: (iii) *x*, 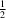 − *y*, − 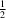 + *z*], are 7.93804 (18) Å (Figs. 3 ▸ and 4 ▸). The nitrate ions coordinating to Zn1 and Zn2 have an N⋯N distance of 3.409 (4) Å (Fig. 3 ▸).

## Synthesis and crystallization

Under an argon atmosphere, zinc nitrate hexa­hydrate (1.5 g, 5 mmol), dissolved in dry methanol (5 ml), was added to a 20 ml dry methano­lic solution of cyclam (1.0 g, 5 mmol). The reaction mixture was agitated at room temperature for 2 h before the solvent was evaporated to get a colorless solid. To obtain colorless crystals appropriate for X-ray crystallography, the crude product was dissolved in hot methanol, filtered through a cellulose filter (0.45 µm pore size) and cooled to room temperature (yield 1.7 g, 87%).

## Refinement

Table 2 ▸ summarizes crystal data, data collection, and structure refinement details.

## Supplementary Material

Crystal structure: contains datablock(s) I. DOI: 10.1107/S2414314622008549/wm4171sup1.cif

Structure factors: contains datablock(s) I. DOI: 10.1107/S2414314622008549/wm4171Isup2.hkl

CCDC reference: 2193206

Additional supporting information:  crystallographic information; 3D view; checkCIF report

## Figures and Tables

**Figure 4:**
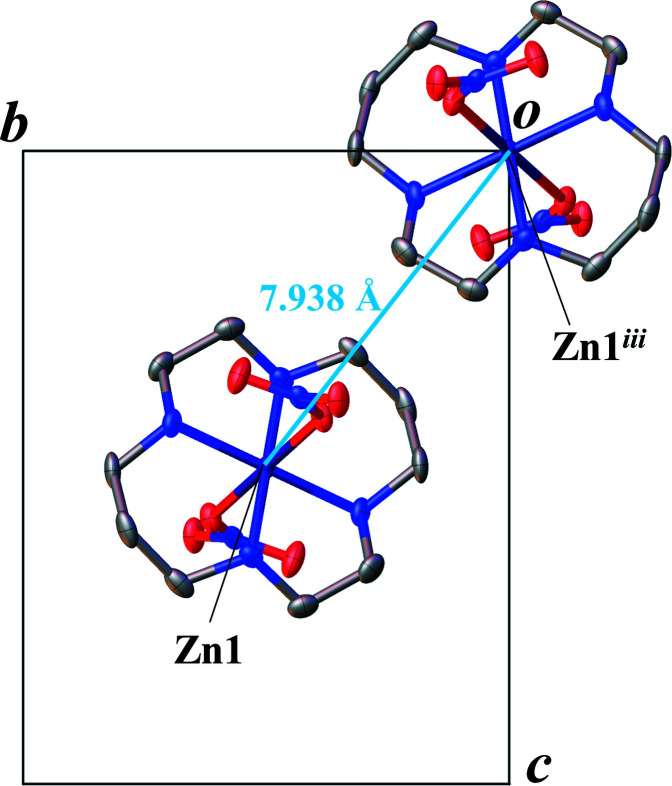
Packing view down the *a* axis of the title complex with displacement ellipsoids drawn at the 50% probability level. Solvent mol­ecules and H atoms are omitted for clarity. [Symmetry code: (iii) *x*, 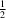 − *y*, − 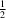 + *z*].

**Table 1:** Hydrogen-bond geometry (Å, °)

*D*—H⋯*A*	*D*—H	H⋯*A*	*D*⋯*A*	*D*—H⋯*A*
N1—H1⋯O2	1.00	2.08	2.995 (3)	151
N2—H2⋯O5^i^	1.00	2.60	3.497 (4)	149
N2—H2⋯O6^i^	1.00	2.14	3.036 (4)	148
N3—H3⋯O5	1.00	2.06	2.931 (3)	145
N4—H4⋯O3	1.00	2.06	2.977 (3)	152
O7—H7⋯O1	0.86	2.38	3.144 (3)	148
O7—H7⋯O3	0.86	2.18	2.966 (3)	151

Symmetry code: (i) 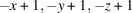 .

**Table 2:** Experimental details

Crystal data	
Chemical formula	[Zn(NO_3_)_2_(C_10_H_24_N_4_)]·CH_3_OH
*M*_r_	421.76
Crystal system, space group	Monoclinic, *P*2_1_/*c*
Temperature (K)	100
*a*, *b*, *c* (Å)	15.3412 (5), 9.4306 (3), 12.7716 (4)
β (°)	105.864 (4)
*V* (Å^3^)	1777.38 (10)
*Z*	4
Radiation type	Cu *K*α
μ (mm^−1^)	2.36
Crystal size (mm)	0.54 × 0.19 × 0.09

Data collection	
Diffractometer	Rigaku XtaLAB Synergy-i
Absorption correction	Multi-scan (*CrysAlis PRO*; Rigaku OD, 2022 ▸)
*T*_min_, *T*_max_	0.356, 1.000
No. of measured, independent and observed [*I* > 2σ(*I*)] reflections	9899, 3230, 2568
*R*_int_	0.078
(sin θ/λ)_max_ (Å^−1^)	0.603

Refinement	
*R*[*F* ^2^ > 2σ(*F* ^2^)], *wR*(*F* ^2^), *S*	0.063, 0.188, 1.01
No. of reflections	3230
No. of parameters	231
H-atom treatment	H-atom parameters constrained
Δρ_max_, Δρ_min_ (e Å^−3^)	1.01, −0.92

Computer programs: *CrysAlis PRO* (Rigaku OD, 2022 ▸), *SHELXT* (Sheldrick, 2015*a* ▸), *SHELXL2018/3* (Sheldrick, 2015*b* ▸) and *OLEX2* (Dolomanov *et al.*, 2009 ▸).

## full crystallographic data

### Crystal data

**Table:**

[Zn(NO_3_)_2_(C_10_H_24_N_4_)]·CH_4_O	*F*(000) = 888
*M**_r_* = 421.76	*D*_x_ = 1.576 Mg m^−^^3^
Monoclinic, *P*2_1_/*c*	Cu *K*α radiation, λ = 1.54184 Å
*a* = 15.3412 (5) Å	Cell parameters from 4300 reflections
*b* = 9.4306 (3) Å	θ = 3.0–68.2°
*c* = 12.7716 (4) Å	µ = 2.36 mm^−^^1^
β = 105.864 (4)°	*T* = 100 K
*V* = 1777.38 (10) Å^3^	Block, clear colourless
*Z* = 4	0.54 × 0.19 × 0.09 mm

### Data collection

**Table:**

Rigaku XtaLAB Synergy-i diffractometer	2568 reflections with *I* > 2σ(*I*)
Detector resolution: 10.0 pixels mm^-1^	*R*_int_ = 0.078
ω scans	θ_max_ = 68.3°, θ_min_ = 3.0°
Absorption correction: multi-scan (*CrysAlisPro*; Rigaku OD, 2022)	*h* = −11→18
*T*_min_ = 0.356, *T*_max_ = 1.000	*k* = −11→11
9899 measured reflections	*l* = −15→15
3230 independent reflections	

### Refinement

**Table:**

Refinement on *F*^2^	Primary atom site location: dual
Least-squares matrix: full	Hydrogen site location: mixed
*R*[*F*^2^ > 2σ(*F*^2^)] = 0.063	H-atom parameters constrained
*wR*(*F*^2^) = 0.188	*w* = 1/[σ^2^(*F*_o_^2^) + (0.1278*P*)^2^] where *P* = (*F*_o_^2^ + 2*F*_c_^2^)/3
*S* = 1.01	(Δ/σ)_max_ = 0.001
3230 reflections	Δρ_max_ = 1.01 e Å^−^^3^
231 parameters	Δρ_min_ = −0.92 e Å^−^^3^
0 restraints	

### Special details

**Table:**

Geometry. All esds (except the esd in the dihedral angle between two l.s. planes) are estimated using the full covariance matrix. The cell esds are taken into account individually in the estimation of esds in distances, angles and torsion angles; correlations between esds in cell parameters are only used when they are defined by crystal symmetry. An approximate (isotropic) treatment of cell esds is used for estimating esds involving l.s. planes.
Refinement. All hydrogen atoms were placed using a geometrical computation.

### Fractional atomic coordinates and isotropic or equivalent isotropic displacement parameters (Å^2^)

**Table:**

	*x*	*y*	*z*	*U*_iso_*/*U*_eq_	
Zn1	1.000000	0.500000	0.500000	0.0174 (3)	
Zn2	0.500000	0.500000	0.500000	0.0210 (3)	
O1	0.90165 (12)	0.6144 (2)	0.57953 (17)	0.0228 (5)	
O4	0.41074 (12)	0.4107 (2)	0.60524 (17)	0.0240 (5)	
O3	0.77063 (11)	0.6485 (2)	0.60726 (18)	0.0285 (6)	
O2	0.83570 (14)	0.4444 (3)	0.6451 (2)	0.0327 (6)	
N4	0.60866 (19)	0.5006 (2)	0.6400 (3)	0.0180 (7)	
H4	0.664921	0.519254	0.617093	0.022*	
O7	0.81007 (13)	0.9063 (2)	0.4951 (2)	0.0361 (6)	
H7	0.812347	0.819336	0.516078	0.043*	
O6	0.28873 (13)	0.3907 (2)	0.6578 (2)	0.0392 (7)	
O5	0.31960 (14)	0.5903 (2)	0.5959 (2)	0.0373 (7)	
N2	0.89281 (18)	0.4685 (3)	0.3604 (2)	0.0212 (6)	
H2	0.835219	0.491792	0.379058	0.025*	
N1	0.98381 (15)	0.3061 (3)	0.5702 (2)	0.0214 (6)	
H1	0.933487	0.318926	0.605250	0.026*	
N5	0.83550 (14)	0.5680 (3)	0.6110 (2)	0.0174 (6)	
N6	0.33879 (16)	0.4640 (3)	0.6198 (2)	0.0203 (6)	
N3	0.47857 (15)	0.7086 (2)	0.5418 (2)	0.0206 (6)	
H3	0.428178	0.705969	0.577575	0.025*	
C9	0.62304 (18)	0.3673 (3)	0.7042 (2)	0.0228 (7)	
H9A	0.570572	0.350872	0.733703	0.027*	
H9B	0.677734	0.377161	0.766423	0.027*	
C5	0.90590 (18)	0.5758 (3)	0.2809 (2)	0.0273 (7)	
H5A	0.849116	0.587691	0.221956	0.033*	
H5B	0.954085	0.544320	0.248060	0.033*	
C8	0.59354 (18)	0.6245 (3)	0.7040 (2)	0.0241 (7)	
H8A	0.650488	0.649443	0.759304	0.029*	
H8B	0.547153	0.601449	0.742138	0.029*	
C6	0.45112 (18)	0.8098 (3)	0.4500 (2)	0.0243 (7)	
H6A	0.501178	0.820272	0.415422	0.029*	
H6B	0.439699	0.903787	0.478126	0.029*	
C7	0.56169 (17)	0.7496 (3)	0.6268 (2)	0.0242 (7)	
H7A	0.548869	0.832283	0.668051	0.029*	
H7B	0.609813	0.776730	0.592444	0.029*	
C1	1.06750 (19)	0.2846 (3)	0.6595 (3)	0.0286 (7)	
H1A	1.116932	0.251144	0.629517	0.034*	
H1B	1.057015	0.211822	0.710621	0.034*	
C10	0.63455 (18)	0.2400 (3)	0.6356 (2)	0.0243 (7)	
H10A	0.658080	0.159721	0.685250	0.029*	
H10B	0.681107	0.263706	0.597912	0.029*	
C2	0.95855 (19)	0.1838 (3)	0.4955 (3)	0.0294 (8)	
H2A	0.947341	0.100243	0.536938	0.035*	
H2B	1.009659	0.160697	0.465025	0.035*	
C4	0.88514 (18)	0.3207 (3)	0.3175 (3)	0.0298 (8)	
H4A	0.940106	0.297254	0.294672	0.036*	
H4B	0.832302	0.314258	0.252673	0.036*	
C3	0.87418 (19)	0.2135 (3)	0.4024 (3)	0.0316 (8)	
H3A	0.853153	0.122895	0.364889	0.038*	
H3B	0.825874	0.247888	0.433994	0.038*	
C11	0.7259 (2)	0.9615 (4)	0.5002 (3)	0.0329 (7)	
H11A	0.676872	0.901659	0.457126	0.049*	
H11B	0.718800	1.058159	0.470901	0.049*	
H11C	0.723491	0.963070	0.576083	0.049*	

### Atomic displacement parameters (Å^2^)

**Table:**

	*U*^11^	*U*^22^	*U*^33^	*U*^12^	*U*^13^	*U*^23^
Zn1	0.0152 (4)	0.0120 (4)	0.0215 (4)	−0.00113 (18)	−0.0010 (3)	0.0007 (2)
Zn2	0.0191 (4)	0.0141 (5)	0.0245 (4)	0.00029 (19)	−0.0030 (3)	−0.0013 (2)
O1	0.0144 (9)	0.0192 (11)	0.0366 (12)	−0.0024 (8)	0.0099 (8)	−0.0047 (9)
O4	0.0131 (9)	0.0232 (12)	0.0364 (11)	0.0042 (8)	0.0080 (8)	0.0045 (10)
O3	0.0145 (9)	0.0193 (12)	0.0517 (14)	0.0038 (8)	0.0093 (9)	−0.0010 (10)
O2	0.0258 (11)	0.0225 (13)	0.0545 (16)	0.0051 (10)	0.0188 (11)	0.0139 (12)
N4	0.0137 (12)	0.0149 (15)	0.0229 (15)	−0.0021 (8)	0.0010 (11)	−0.0007 (9)
O7	0.0321 (11)	0.0227 (13)	0.0584 (16)	0.0018 (9)	0.0207 (11)	0.0021 (12)
O6	0.0214 (10)	0.0341 (14)	0.0685 (17)	0.0064 (10)	0.0229 (11)	0.0227 (13)
O5	0.0291 (11)	0.0165 (13)	0.0733 (19)	0.0070 (10)	0.0258 (12)	0.0078 (12)
N2	0.0146 (12)	0.0234 (13)	0.0230 (15)	0.0013 (11)	0.0006 (10)	−0.0039 (12)
N1	0.0165 (11)	0.0156 (13)	0.0339 (14)	0.0035 (9)	0.0102 (10)	0.0027 (11)
N5	0.0095 (10)	0.0179 (14)	0.0224 (12)	−0.0002 (10)	0.0002 (9)	−0.0036 (11)
N6	0.0106 (11)	0.0263 (15)	0.0211 (13)	0.0018 (12)	−0.0007 (9)	0.0019 (12)
N3	0.0162 (11)	0.0163 (13)	0.0283 (13)	−0.0028 (9)	0.0045 (9)	−0.0011 (10)
C9	0.0156 (12)	0.0218 (17)	0.0293 (15)	0.0014 (12)	0.0036 (11)	0.0049 (13)
C5	0.0182 (13)	0.039 (2)	0.0228 (15)	0.0049 (13)	0.0017 (11)	0.0058 (14)
C8	0.0189 (13)	0.0247 (18)	0.0267 (15)	−0.0022 (12)	0.0030 (11)	−0.0063 (14)
C6	0.0166 (13)	0.0168 (15)	0.0376 (17)	−0.0011 (11)	0.0042 (11)	0.0004 (14)
C7	0.0183 (13)	0.0202 (16)	0.0319 (16)	−0.0049 (12)	0.0030 (11)	−0.0059 (14)
C1	0.0208 (14)	0.0287 (19)	0.0354 (18)	0.0094 (13)	0.0059 (12)	0.0119 (15)
C10	0.0172 (12)	0.0186 (16)	0.0331 (16)	0.0021 (12)	0.0001 (11)	0.0036 (14)
C2	0.0238 (15)	0.0110 (15)	0.055 (2)	−0.0010 (12)	0.0142 (14)	−0.0033 (14)
C4	0.0153 (13)	0.036 (2)	0.0357 (18)	−0.0025 (14)	0.0035 (12)	−0.0174 (16)
C3	0.0178 (13)	0.0199 (17)	0.057 (2)	−0.0060 (12)	0.0104 (13)	−0.0149 (15)
C11	0.0259 (13)	0.0356 (19)	0.0372 (19)	0.0017 (17)	0.0084 (12)	0.0016 (16)

### Geometric parameters (Å, º)

**Table:**

Zn1—O1	2.3045 (18)	N3—C7	1.483 (3)
Zn1—O1^i^	2.3045 (18)	C9—H9A	0.9900
Zn1—N2	2.090 (3)	C9—H9B	0.9900
Zn1—N2^i^	2.090 (3)	C9—C10	1.524 (4)
Zn1—N1^i^	2.081 (2)	C5—H5A	0.9900
Zn1—N1	2.081 (2)	C5—H5B	0.9900
Zn2—O4^ii^	2.3233 (19)	C5—C1^i^	1.520 (4)
Zn2—O4	2.3232 (19)	C8—H8A	0.9900
Zn2—N4^ii^	2.085 (3)	C8—H8B	0.9900
Zn2—N4	2.085 (3)	C8—C7	1.530 (4)
Zn2—N3	2.087 (2)	C6—H6A	0.9900
Zn2—N3^ii^	2.087 (2)	C6—H6B	0.9900
O1—N5	1.267 (3)	C6—C10^ii^	1.535 (4)
O4—N6	1.272 (3)	C7—H7A	0.9900
O3—N5	1.242 (3)	C7—H7B	0.9900
O2—N5	1.244 (4)	C1—H1A	0.9900
N4—H4	1.0000	C1—H1B	0.9900
N4—C9	1.484 (4)	C10—H10A	0.9900
N4—C8	1.480 (4)	C10—H10B	0.9900
O7—H7	0.8603	C2—H2A	0.9900
O7—C11	1.410 (4)	C2—H2B	0.9900
O6—N6	1.228 (3)	C2—C3	1.525 (4)
O5—N6	1.245 (4)	C4—H4A	0.9900
N2—H2	1.0000	C4—H4B	0.9900
N2—C5	1.485 (4)	C4—C3	1.525 (5)
N2—C4	1.490 (4)	C3—H3A	0.9900
N1—H1	1.0000	C3—H3B	0.9900
N1—C1	1.480 (4)	C11—H11A	0.9800
N1—C2	1.480 (4)	C11—H11B	0.9800
N3—H3	1.0000	C11—H11C	0.9800
N3—C6	1.481 (4)		
			
O1^i^—Zn1—O1	180.0	N4—C9—C10	111.9 (2)
N2^i^—Zn1—O1	90.86 (9)	H9A—C9—H9B	107.9
N2—Zn1—O1^i^	90.86 (9)	C10—C9—H9A	109.2
N2—Zn1—O1	89.14 (9)	C10—C9—H9B	109.2
N2^i^—Zn1—O1^i^	89.14 (9)	N2—C5—H5A	110.0
N2—Zn1—N2^i^	180.00 (15)	N2—C5—H5B	110.0
N1^i^—Zn1—O1^i^	92.98 (8)	N2—C5—C1^i^	108.4 (2)
N1^i^—Zn1—O1	87.02 (8)	H5A—C5—H5B	108.4
N1—Zn1—O1^i^	87.02 (8)	C1^i^—C5—H5A	110.0
N1—Zn1—O1	92.98 (8)	C1^i^—C5—H5B	110.0
N1^i^—Zn1—N2^i^	94.81 (10)	N4—C8—H8A	109.9
N1—Zn1—N2^i^	85.19 (10)	N4—C8—H8B	109.9
N1^i^—Zn1—N2	85.19 (10)	N4—C8—C7	108.9 (2)
N1—Zn1—N2	94.81 (10)	H8A—C8—H8B	108.3
N1—Zn1—N1^i^	180.00 (12)	C7—C8—H8A	109.9
O4—Zn2—O4^ii^	180.0	C7—C8—H8B	109.9
N4^ii^—Zn2—O4^ii^	87.95 (9)	N3—C6—H6A	109.3
N4—Zn2—O4^ii^	92.05 (9)	N3—C6—H6B	109.3
N4^ii^—Zn2—O4	92.05 (9)	N3—C6—C10^ii^	111.7 (2)
N4—Zn2—O4	87.95 (9)	H6A—C6—H6B	107.9
N4^ii^—Zn2—N4	180.0	C10^ii^—C6—H6A	109.3
N4^ii^—Zn2—N3	94.42 (9)	C10^ii^—C6—H6B	109.3
N4—Zn2—N3^ii^	94.42 (9)	N3—C7—C8	109.2 (2)
N4^ii^—Zn2—N3^ii^	85.58 (9)	N3—C7—H7A	109.8
N4—Zn2—N3	85.58 (9)	N3—C7—H7B	109.8
N3^ii^—Zn2—O4	88.02 (8)	C8—C7—H7A	109.8
N3—Zn2—O4	91.98 (8)	C8—C7—H7B	109.8
N3^ii^—Zn2—O4^ii^	91.98 (8)	H7A—C7—H7B	108.3
N3—Zn2—O4^ii^	88.02 (8)	N1—C1—C5^i^	108.9 (2)
N3—Zn2—N3^ii^	180.0	N1—C1—H1A	109.9
N5—O1—Zn1	130.97 (17)	N1—C1—H1B	109.9
N6—O4—Zn2	127.85 (18)	C5^i^—C1—H1A	109.9
Zn2—N4—H4	107.5	C5^i^—C1—H1B	109.9
C9—N4—Zn2	115.77 (18)	H1A—C1—H1B	108.3
C9—N4—H4	107.5	C9—C10—C6^ii^	116.0 (2)
C8—N4—Zn2	105.45 (18)	C9—C10—H10A	108.3
C8—N4—H4	107.5	C9—C10—H10B	108.3
C8—N4—C9	112.7 (3)	C6^ii^—C10—H10A	108.3
C11—O7—H7	107.3	C6^ii^—C10—H10B	108.3
Zn1—N2—H2	107.8	H10A—C10—H10B	107.4
C5—N2—Zn1	105.34 (18)	N1—C2—H2A	109.2
C5—N2—H2	107.8	N1—C2—H2B	109.2
C5—N2—C4	113.4 (3)	N1—C2—C3	112.1 (2)
C4—N2—Zn1	114.27 (19)	H2A—C2—H2B	107.9
C4—N2—H2	107.8	C3—C2—H2A	109.2
Zn1—N1—H1	106.5	C3—C2—H2B	109.2
C1—N1—Zn1	105.80 (17)	N2—C4—H4A	109.3
C1—N1—H1	106.5	N2—C4—H4B	109.3
C2—N1—Zn1	116.65 (18)	N2—C4—C3	111.8 (3)
C2—N1—H1	106.5	H4A—C4—H4B	107.9
C2—N1—C1	114.1 (2)	C3—C4—H4A	109.3
O3—N5—O1	118.6 (2)	C3—C4—H4B	109.3
O3—N5—O2	120.8 (2)	C2—C3—H3A	108.2
O2—N5—O1	120.6 (2)	C2—C3—H3B	108.2
O6—N6—O4	119.8 (3)	C4—C3—C2	116.2 (2)
O6—N6—O5	120.3 (2)	C4—C3—H3A	108.2
O5—N6—O4	119.9 (2)	C4—C3—H3B	108.2
Zn2—N3—H3	106.8	H3A—C3—H3B	107.4
C6—N3—Zn2	115.81 (18)	O7—C11—H11A	109.5
C6—N3—H3	106.8	O7—C11—H11B	109.5
C6—N3—C7	114.4 (2)	O7—C11—H11C	109.5
C7—N3—Zn2	105.63 (17)	H11A—C11—H11B	109.5
C7—N3—H3	106.8	H11A—C11—H11C	109.5
N4—C9—H9A	109.2	H11B—C11—H11C	109.5
N4—C9—H9B	109.2		
			
Zn1—O1—N5—O3	−149.2 (2)	N4—C9—C10—C6^ii^	−71.3 (3)
Zn1—O1—N5—O2	30.9 (4)	N4—C8—C7—N3	57.2 (3)
Zn1—N2—C5—C1^i^	−42.5 (2)	N2—C4—C3—C2	−73.0 (3)
Zn1—N2—C4—C3	57.8 (3)	N1—C2—C3—C4	70.0 (3)
Zn1—N1—C1—C5^i^	41.5 (2)	C9—N4—C8—C7	−169.3 (2)
Zn1—N1—C2—C3	−53.7 (3)	C5—N2—C4—C3	178.6 (2)
Zn2—O4—N6—O6	164.3 (2)	C8—N4—C9—C10	177.6 (2)
Zn2—O4—N6—O5	−16.1 (4)	C6—N3—C7—C8	−168.6 (2)
Zn2—N4—C9—C10	56.1 (3)	C7—N3—C6—C10^ii^	179.3 (2)
Zn2—N4—C8—C7	−42.1 (2)	C1—N1—C2—C3	−177.7 (2)
Zn2—N3—C6—C10^ii^	56.1 (3)	C2—N1—C1—C5^i^	171.1 (2)
Zn2—N3—C7—C8	−40.1 (2)	C4—N2—C5—C1^i^	−168.2 (2)

Symmetry codes: (i) −*x*+2, −*y*+1, −*z*+1; (ii) −*x*+1, −*y*+1, −*z*+1.

### Hydrogen-bond geometry (Å, º)

**Table:**

*D*—H···*A*	*D*—H	H···*A*	*D*···*A*	*D*—H···*A*
N1—H1···O2	1.00	2.08	2.995 (3)	151
N2—H2···O5^ii^	1.00	2.60	3.497 (4)	149
N2—H2···O6^ii^	1.00	2.14	3.036 (4)	148
N3—H3···O5	1.00	2.06	2.931 (3)	145
N4—H4···O3	1.00	2.06	2.977 (3)	152
O7—H7···O1	0.86	2.38	3.144 (3)	148
O7—H7···O3	0.86	2.18	2.966 (3)	151

Symmetry code: (ii) −*x*+1, −*y*+1, −*z*+1.
